# Clinical characteristics and outcomes for children, adolescents and young adults with “CIC‐fused” or “BCOR‐rearranged” soft tissue sarcomas: A multi‐institutional European retrospective analysis

**DOI:** 10.1002/cam4.6113

**Published:** 2023-05-22

**Authors:** Monika Sparber‐Sauer, Nadège Corradini, Maria Carmen Affinita, Giuseppe Maria Milano, Gaelle Pierron, Matthieu Carton, Franck Tirode, Daniel Pissaloux, Rita Alaggio, Christian Vokuhl, Gianni Bisogno, Pablo Berlanga, Andrea Ferrari, Daniel Orbach

**Affiliations:** ^1^ Klinikum der Landeshauptstadt Stuttgart gKAöR, Olgahospital, Stuttgart Cancer Center, Zentrum für Kinder‐, Jugend‐ und Frauenmedizin, Pädiatrie 5 (Pädiatrische Onkologie, Hämatologie, Immunologie) Stuttgart Germany; ^2^ Medizinische Fakultät der Universität Tübingen Tübingen Germany; ^3^ Department of Pediatric Hematology and Oncology‐IHOPe Léon Bérard Center Lyon France; ^4^ Pediatric Hematology, Oncology and Stem Cell Transplant Division Department of Women's and Children's Health University Hospital of Padova Padova Italy; ^5^ Department of Hematology and Oncology, Cell and Gene Therapy Bambino Gesù Children's Hospital, IRCCS Rome Italy; ^6^ Department of Somatic Genetics Institut Curie Paris France; ^7^ Department of Biometry Institut Curie, DRCI, PSL Research University Paris France; ^8^ Department of Biopathology Centre Léon Bérard Lyon France; ^9^ INSERM U1052, Cancer Research Centre of Lyon Lyon France; ^10^ Pathology Unit, Department of Laboratories Bambino Gesù Children's Hospital, IRCCS Rome Italy; ^11^ Section of Pediatric Pathology, Department of Pathology Bonn Germany; ^12^ Department of Pediatric and Adolescent Oncology, Gustave Roussy Cancer Campus Université Paris‐Saclay Villejuif France; ^13^ Pediatric Oncology Unit Fondazione IRCCS Istituto Nazionale Tumori Milan Italy; ^14^ SIREDO Oncology Center (Care, Innovation and Research for Children, Adolescents and Young Adults with Cancer) Institut Curie, PSL University Paris France

**Keywords:** *BCOR* rearrangement, *CIC‐DUX* fusion transcript, pediatric/adolescent oncology, soft tissue sarcoma, *YWHAE* fusion transcript

## Abstract

**Background:**

In certain rare undifferentiated small round cell sarcomas new specific molecular *CIC‐DUX4*/other partner, *BCOR‐CCNB3*/other partner, *YWHAE* fusions, or *BCOR‐ITD* (internal tandem duplication) were identified. These new “*CIC fused*” (CIC‐fused/*ATXN1::NUTM1*) and “*BCOR rearranged*” (BCOR fused/ITD/ *YWHAE*) soft tissue sarcomas (STS) are not well described.

**Methods:**

Multi‐institutional European retrospective analysis of young patients (0–24 years) with CIC‐fused and BCOR rearranged STS.

**Results:**

Overall, out of the 60 patients selected, the fusion status was CIC‐fused (*n* = 29), *ATXN1::NUTM1* (*n* = 2), *BCOR::CCNB3* (*n* = 18), BCOR‐ITD (*n* = 7), and YWHAE (*n* = 3), *MAML::BCOR* STS (*n* = 1). The main primaries were abdomen‐pelvic (*n* = 23) and limbs (*n* = 18). Median age was 14 years (0.9–23.8) and 0.9 (0.1–19.1) for CIC‐fused and BCOR‐rearranged groups, respectively (*n* = 29; *p* < 0.001). IRS stages were I (*n* = 3), II (*n* = 7), III (*n* = 35), and IV (*n* = 15). Overall, 42 patients had large tumors (>5 cm) but only six had lymph node involvement. Patients received mainly chemotherapy (*n* = 57), local surgery (*n* = 50), and/or radiotherapy (*n* = 34). After a median follow‐up of 47.1 months (range, 3.4–230), 33 (52%) patients had an event and 23 patients died. Three‐year event‐free survivals were 44.0% (95% CI 28.7–67.5) and 41.2% (95% CI 25.4–67.0) for CIC and BCOR groups (*p* = 0.97), respectively. Three‐year overall survivals were 46.3% (95% CI 29.6–72.4) and 67.1% (95% CI 50.4–89.3; *p* = 0.24), respectively.

**Conclusions:**

Pediatric patients often present with large tumors and metastatic disease, especially CIC sarcomas. Overall outcome is dismal. New treatment options are needed.

## Abstract 2

1. CIC, BCOR, and YWHAE rearranged soft tissue sarcomas are poorly characterized.
2. Analysis of 60 young patients with these very rare sarcomas.
3. Overall outcome is dismal and new therapies are warranted.

## 1 INTRODUCTION

A new subset of round/ovoid cell sarcomas histologically resembling tumors of the Ewing sarcoma family (ESFT) has been recently characterized in unclassified/undifferentiated small round cell sarcoma (USRCS or SRCSs).^1^, ^2^, ^3^, ^4^, ^5^, ^6^ Previously known as “Ewing‐like sarcomas” (ELS), these tumors are currently distinguished from Ewing sarcoma in the current WHO classification. With molecular assays these tumors have been genetically characterized and are now under assessment if these entities may represent distinct biologic tumor entities.^7^, ^8^, ^9^, ^10^ Accordingly, a new subtype of bone sarcoma has been characterized by a *BCOR::CCNB3* gene fusion.^11^ This discovery offers new insight into oncogenesis and future new therapeutic considerations. Rare *BCOR* sarcoma was described in a group of small round cell bone tumors lacking the canonical EWSR1 translocation: only 24 *BCOR::CCNB3* positive tumors could be identified in an analysis of 594 sarcoma.^11^ Other “*BCOR* rearranged sarcomas” have been described with molecular characteristics as *BCOR‐ITD* (internal tandem duplication).^12^ These *BCOR‐ITD* tumors are reported to be aggressive tumor entities with an ITD in the last exon of the *BCOR* gene. With location in the kidney, these sarcomas are described as clear cell sarcomas of the kidney (CCSK).^13^ However, these sarcomas are also described as high‐grade endometrial stromal sarcomas (HG‐ESS)^14^, ^15^ in bone and soft tissue, referred to as USRCS, but also occurring in infants as primitive mesenchymal myxoid tumor (PMMTI).^16^, ^17^, ^18^ *CIC::DUX4* sarcoma (CDS) or CIC‐rearranged sarcomas seem a rare subcategory of another small round cell sarcoma with histological signs of Ewing sarcoma (ES).^2^ For extracranial sites, “*CIC* fused sarcoma” group had biological homology as the “*BCOR* rearranged sarcoma” group.^6^ In recent analysis, *ATXN1* tumors clustered with CIC tumors and *YWHAE* fused tumors within the BCOR tumors group. For the *BCOR‐ITD* tumors no radiological, clinical, nor pathological specificity could be found, except for location in the central nervous system but a common transcriptomic signature with other *BCOR* rearranged sarcomas.^18^ However, all these are described as a distinct entity from ES, and occur in various sites as soft tissue, visceral primaries and less often bone.^3^, ^19^

Therapeutic options for such tumors frequently include chemotherapy (CHT) and local surgery with radiotherapy (RT) analogous to treatment of soft tissue sarcomas or ES.^19^ There are few data available regarding the clinical description of these tumors when the primary site is soft tissue. We describe the clinical characteristics, treatment, outcomes, and prognostic factors of an European cohort of pediatric and young patients with *CIC*, *BCOR*, or *YWHAE* gene rearranged soft tissue sarcomas.

## 2 METHODS

This retrospective multi‐institutional study registered all patients aged ≤24 years at the time of diagnosis with USRCS in soft tissues identified from local coordinators as *CIC*, *BCOR*, or *YWHAE* gene rearranged sarcomas. Location in bone, central nervous system, or kidney were exclusion criteria. Patients diagnosed between 1996 and 2021 with available clinical data and at least 3 months of follow‐up were included. All specimen were reviewed by expert pathologists or by central review for those that were enrolled on clinical trials. Available molecular data were mandatory for all cases.^6^ In clinical practice, diagnosis of the oncogenic fusion was performed using molecular techniques such as fluorescence in situ hybridization (FISH), reverse transcription polymerase chain reaction (RT‐PCR), or targeted RNA sequencing.^6^ Depending on tumor tissue availability, RT‐PCR, next‐generation RNA sequencing (RNA seq) or clustering, and methylation profile were performed on paraffin‐embedded and/or fresh frozen tissue.^2^, ^4^, ^20^ Recently, all cases of tumors with pathological Ewing characteristics that do not harbor the canonic EWS fusion transcript, were systematically analyzed with RNA sequencing and/or RT‐PCR with specific combination of primer sets for *CIC::DUX4* and *BCOR* and/or FISH analysis with probes for *CIC (19q13.2)*, for *BCOR* a dual color break apart probe (Xp 11.4) and for *YWHEA* (17p13.3).^11^, ^21^, ^22^ The order of this techniques varies according to each institutional organization. For some old cases, analysis could have been performed retrospectively.

Due to their biological homology, all sarcomas with *CIC::DUX4*, *CIC* break, or *ATXN1::NUTM1* fusion transcript were gathered in the “*CIC* fused sarcoma” group and sarcomas with *BCOR::CCNB3*, BCOR‐ITD, *YWHAE::NUTM2B*, or *MAML::BCOR* fusion transcripts represent the “*BCOR* rearranged sarcoma” group.^6^

Guardians of included patients had consented to data collection and retrospective chart review. This was performed per the requirements of the Declaration of Helsinki and in accordance with the regulations of the respective ethical committee (Ethikkommission Tübingen 413/2022BO2 from August 18, 2022).

### 2.1 Definition of terms

Clinical staging was defined according to the Tumor Node Metastases (TNM) system: T1 or T2 according to the invasion of contiguous organs; N0/N1 according to the presence of lymph node metastases and M0/M1 of distant metastases.^23^ Postsurgical staging was classified according to the International Rhabdomyosarcoma Study grouping (IRS‐G) system. IRS‐I corresponds to a complete tumor resection with microscopically complete margins, IRS‐II corresponds to a macroscopic resection but invaded histologic margins and IRS‐III corresponds to a macroscopic residual tumor after biopsy or immediate surgery. IRS‐IV corresponds to metastatic disease.^24^ Initial staging included imaging of the primary tumor and bone/lung metastases by magnetic resonance imaging (MRI) or computed tomography (CT). Whole body imaging was recommended with radionuclide bone scan or 18F‐fluorodeoxyglucose positron emission tomography. Staging included bone marrow aspirate/biopsy. Tumor response was mainly assessed after 3–4 courses of CHT: complete response (CR), partial response (PR), or stable disease (SD).^25^ Delayed surgical resection was defined if the resection took part after initiation of chemotherapy. “Extent of tumor resection” was defined as the best surgical result in the sum of any surgeries that were performed in one patient during the first‐line therapy. Primary tumor resection was classified as microscopically complete (R0), microscopically incomplete (R1), or macroscopically incomplete (R2).^26^ “Best tumor response” was the best available response at any time point after start chemotherapy. Progressive disease (PD) was defined as first event with in case of any increase in tumor volume in patients who did not achieve CR.^27^

### 2.2 Therapy

No dedicated protocols were available and the treatment strategy the individual decision of the treating physicians. Patients were classically treated according to Ewing's or soft tissue sarcoma (STS)/ rhabdomyosarcoma (RMS) protocols, with a combination of therapies including neoadjuvant and adjuvant CHT, surgical resection, and/or radiation therapy (RT) of the primary according to initial tumor extension and the response to initial chemotherapy.^28^ Primary resection was performed if a non‐mutilating procedure was possible. RT was given as individual decision of the treating center.

### 2.3 Statistical analysis

Survival time was measured from the date of diagnosis (initial biopsy/surgery) to the time of last follow‐up or event. Event‐free survival (EFS) was defined as the time from diagnosis to the date of local relapse or any lymph node or metastatic tumor progression or death from any cause. Overall survival (OS) was calculated from the date of biopsy/surgery to death from any cause. Patients alive without local recurrence or metastasis at last contact were censored at the date of last follow‐up. Kaplan–Meier method was used for survival curves and log‐rank test to calculate the differences between survivals. The prognostic role of risk factors (gender, age at diagnosis [± median of the cohort], tumor size [±5 cm] and site, TNM status, IRS groups [IRS I–II vs. III vs. IV] and type of tumor groups [*CIC‐*fused vs. *BCOR* groups]) were considered for their impact on EFS and OS by comparing Kaplan–Meier curves using log‐rank tests and Cox univariate and multivariate models. All risk factors significant at least at *p* 0.25 in univariate analysis were included in the multivariable model; type of tumor group was forced in every model.

## 3 RESULTS

### 3.1 Patients' characteristics and demography

Since 1996, 60 patients fulfilled the inclusion criteria. Patients were treated in France (*n* = 31), Italy (*n* = 18), Germany (*n* = 10), and Switzerland (*n* = 1). Patients´ and tumors' characteristics are presented in Tables 1 and 2. Median age of patients at initial diagnosis with “*CIC* fused sarcoma” was 14 years (range, 0.9–23.8), and 0.9 years (range, 0–19.1) for “*BCOR* rearranged sarcomas”. Primary tumor was mainly located in trunk (53%) and specifically in abdomen/pelvic site (38%). Tumors were frequently large (>5 cm, 72%) and extensive T2 (60%) but only 11% had lymph node involvement. In 58% of patients, the primary tumor was unresectable at diagnosis (IRS‐III, *n* = 35) and metastatic disease was present in 25% (IRS‐IV, *n* = 15). Metastatic patients had mostly pulmonary metastases (*n* = 9/15) and “*CIC*‐fused sarcoma” (*n* = 13/15).

**TABLE 1:** Clinical characteristics of the population with CIC/BCOR rearranged sarcomas.

	CIC‐fused sarcomas 31 cases	BCOR rearranged tumors 29 cases	Total	*p* value
Median age (range) years	14 (0.9–23.8)	0.9 (0–19.1)	60	<0.001
Gender				<0.001
Male	11	23	34	
Female	20	6	26	
Tumor site				0.011
Head and neck	6	4	10	
Limbs	14	4	18	
Trunk (thorax, pelvis, abdomen)	11	21	32	
Tumor size				1
≤5 cm	8	8	16	
>5 cm	21	21	42	
NA	2	0	2	
T status				0.65
T1	10	11	21	
T2	21	18	39	
N status				1
N0	26	25	51	
N1	3	3	6	
NA	2	1	3	
M status				0.002
M0	18	27	45	
M1	13	2	15	
IRS stage				0.006
IRS I–II	3	7	10	
IRS III	15	20	35	
IRS IV	13	2	15	

Abbreviations: IRS, Intergroup Rhabdomyosarcoma Staging; M, metastasis; N, node; NA, not available; R0, complete resection (IRS‐I); R1, incomplete microscopic resection (IRS‐II); R2, macroscopic residue (IRS III); T, tumor.

**TABLE 2:** Tumor characteristics.

	CIC tumors group *N* = 31	BCOR tumors group *N* = 29
Initial pathologic diagnosis		
Undifferentiated small round cells sarcoma	13	6
Other undifferentiated sarcoma	5	9
Ewing‐like sarcoma	9	4
MPNST	1	–
Primitive myxoid mesenchymal tumor of infancy	1	1
Malignant sarcomatoid tumor	–	1
Adamantinoma	1	–
Angiosarcoma	–	4
Congenital fibrosarcoma	–	1
Synovial sarcoma	–	1
Clear cell sarcoma	1	–
Germ cell tumor	–	2
Pathology findings		
Necrosis		
Presence	23	5
Absence	2	10
Not mentioned	6	14
Mitotic rate		
High	17	8
Intermediate/low	5	1
Not done	9	20
Immunohistochemistry		
Nuclear CD99 staining		
Strong	9	3
Absence	17	13
Not done	5	13
Cytoplasmic CD99 staining		
Strong	14	10
Absence	12	6
Not done	5	13
BCOR expression		
Presence	0	6
Absence	11	6^a^
Not done	20	17
Cyclin D1 expression		
Presence		2
Absence		4
Not done		23
Vimentin expression		
Strong	9	3
Absence	–	3
Not done	22	23
Molecular analysis^a^		
FISH	8	6
RT‐PCR/dd PCR	14	4
RNA seq	5	6
NGS	3	1
Methylation profile	2	1
Unknown	1	12
Type of molecular rearrangement	22 *CIC::DUX4*	18 *BCOR::CCNB3*
	7 CIC break (FISH)	7 BCOR‐ITD
	2 *ATXN1::NUTM1*	3 *YWHAE::NUTM2B*
		1 *MAML::BCOR*

Abbreviations: FISH, fluorescence in situ hybridization; N, number; NGS, next‐generation sequencing; RNA seq, RNA sequencing; RT‐PCR, reverse transcriptase polymerase chain reaction; RT‐PCR dd, reverse transcription polymerase chain reaction droplet digital.

^a^ 4 *BCOR::CCNB3*, 1 BCOR‐ITD and 1 *YWHAE::NUTM2B*.

Pathologic diagnosis was mainly USRCS (*n* = 19), other undifferentiated type sarcoma (UDS, *n* = 14), “ELS” (*n* = 13) and various (*n* = 14) (Table 2). Overall, 31 (52%) contained a molecular rearrangement of the “*CIC* fused sarcoma” group and 29 of the “*BCOR* rearranged sarcoma” group. “*CIC* fused sarcoma” group consisted of *CIC::DUX4* (*n* = 22), *CIC* break (FISH) (*n* = 7) and *ATXN1::NUTM1* (*n* = 2). The “*BCOR* rearranged sarcoma” group consisted of *BCOR::CCNB3* (*n* = 18), BCOR‐ITD (*n* = 7), *YWHAE::NUTM2B* (*n* = 3), and *MAML::BCOR* STS (*n* = 1; Table 2).

### 3.2 Treatment

Patients received chemotherapy (*n* = 57), local surgery (*n* = 50), and/or radiotherapy (*n* = 34) (Table 3). Systemic treatment was delivered according to STS/RMS protocols (IVA/VAC, VAIA, IVADo, Ifo‐Doxo; *n* = 33) or Ewing sarcoma protocol (VDC‐IE/VIDE/VAI; *n* = 19). No CHT was administered in three young patients (<3 years old) with localized BCOR rearranged tumor (2 IRS‐II without any other therapy, and 1 IRS‐III with adjuvant radiotherapy). Most patients received alkylating based CHT: ifosfamide (*n* = 27) or cyclophosphamide (*n* = 2) or both (*n* = 15). Anthracyclines based regimen were frequently added (80%), especially for “*CIC* fused sarcomas”. Additional HD CHT, with busulfan and melphalan (*n* = 4) or unspecified regimen (*n* = 1), was delivered in IRS‐III tumors (*n* = 4) and IRS‐IV (*n* = 1). Maintenance treatment with cyclophosphamide/vinorelbine (*n* = 5) or oral trofosfamide/idarubicine/etoposide (*n* = 1) was given (unspecified regimen, *n* = 2). Local primary tumor resection was performed at diagnosis (*n* = 20), as delayed resection after CHT ± RT reduction (*n* = 28) or both (*n* = 2). Most patients with initial unresected/unresectable tumor (IRS group III) could benefit for a R0‐R1 delayed resection in 22/35 cases (63%).

**TABLE 3:** Summary of delivered therapy.

	CIC tumors group *N* = 31	BCOR tumors group *N* = 29	Total *N* = 60	*p* value
Local treatment				0.04
Surgery alone	7	15	22	
Radiation alone	3	3	6	
Both	19	9	28	
None^c^	2	2	4	
Primary tumor resection				1
No	5	5	10	
Yes	26	24	50	
Best tumor resection				0.007
R0	17	7	24	
R1	4	16	17	
R2	5	2	7	
NA	5	7	12	
Chemotherapy regimens				
Anthracyclines based^a^	29	19	48	0.011
Platinum based^a^	3	3	6	1
High dose^b^	2	3	5	1
Maintenance therapy	5	3	8	0.75
Overall chemotherapy regimen				Not performed
According to Ewing protocols (VDCy‐IE/VIDE + VAI)	13	6	19	
According to RMS‐NRSTS protocols (IVA/VAC, VAIA, IVADo, Ifo‐doxo)	15	17	32	
Other regimens	3	3	6	
None	–	3	3	
Radiotherapy				0.021
No	9	17	26	
Yes	22	12	34	

Abbreviations: IE, ifosfamide‐etoposide; Ifo‐doxo, ifosfamide‐doxorubicin; N, number; NA, not available; R0, complete resection; R1, microscopic incomplete resection; R2, macroscopic residue; Rx, data not available; VAC, vincristine‐D actinomycin‐cyclophosphamide; VAI/IVA, vincristine‐D actinomycin‐ifosfamide; VAIA, IVADo, IVA with doxorubicin; VDCy, vincristine‐doxorubicin‐cyclophosphamide; VIDE, vincristine‐ifosfamide‐doxorubicin‐etoposide.

^a^ One missing data.

^b^ 2 missing data.

^c^ 1 toxic death, 3 early progressive disease.

RT of the primary tumor was delivered (*n* = 34): for localized disease (*n* = 26) and metastatic disease (*n* = 8) after best result R0 resection (*n* = 17), R1 resection (*n* = 6) and R2 resection (*n* = 3) (details on resection not available [*n* = 2]), or exclusively (*n* = 6). Median radiation dose at primary tumor was 54 Gray (Gy; range, 42–60). Metastatic sites were irradiated on lung metastases at 18 Gy (*n* = 6; missing data, *n* = 2).

Patients with CIC sarcomas were more likely to receive primary surgery and irradiation, while BCOR tumors were more frequently treated with exclusive surgery (*p* = 0.04, Table 3).

### 3.3 Characteristics according to molecular group

In the cohort of 31 patients with “*CIC* fusion sarcoma”, median age was 14 years (range 0.9–23.8) and metastatic disease was present in 13/31 patients (42%, Table 1, Table S1). Most patients had no regional lymph node involvement (84%), were female (65%) with a primary tumor frequently located in limbs (45%). Maximum tumor size was mainly >5 cm (68%), and extensive (T2, 68%). In contrast, the 29 patients in the “*BCOR* rearranged sarcoma” group were younger (*p* < 0.001) with a median age of 0.9 years overall (0.1–19.1) and specifically 4 years (range, 0.1–19.1) for *BCOR::CCNB3* (*n* = 18) and 1 year (0.3–18.3) for *BCOR‐ITD* (*n* = 7). Localized disease was present in 27/29 patients (93%, *p* = 0.002), with metastasis or nodal involvement only present for two *YWHAE::NUTM2B* fused tumors. Most patients were male (79%, *p* < 0.001), with frequent abdomen/ pelvis primary (72%, *p* = 0.011). Tumor size was also frequently large (>5 cm; 72%) and T2 (62%) (Table 1, Table S2).

Patients with “*CIC* fused sarcoma” had been treated more often with CHT including anthracyclines (94% vs. 66%, respectively; *p* = 0.01), had more complete surgery (R0 vs. R1/R2; 65.4% vs. 31.8%, *p* = 0.007) and received more frequently RT (71% vs. 41%, *p* = 0.02) than patients with “*BCOR* rearranged sarcomas” (Table 3).

### 3.4 Outcome

**FIGURE 1:**
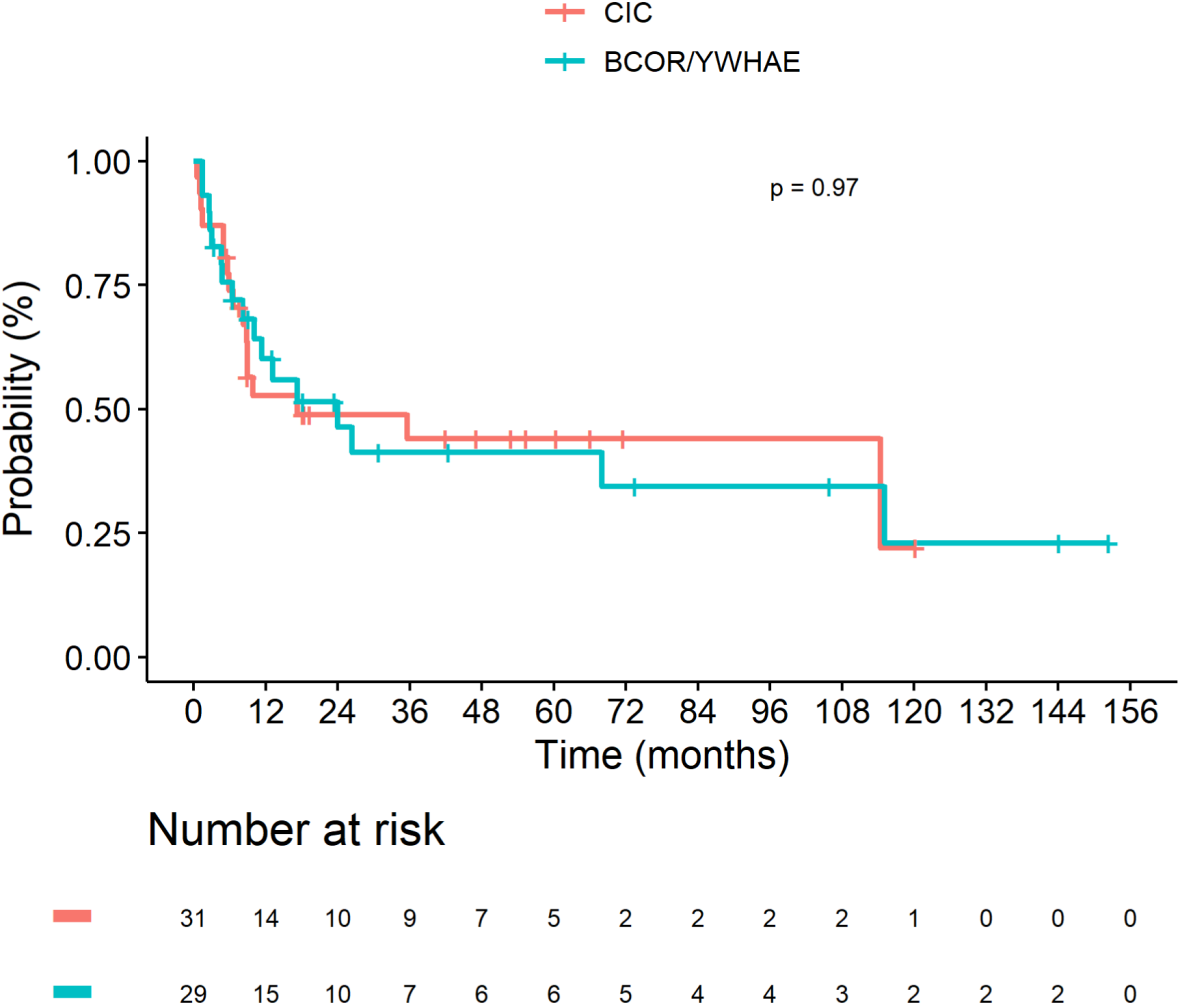
Kaplan–Meier estimates presenting EFS of 60 patients with CIC‐fused sarcoma (*n* = 31) and BCOR rearranged sarcoma (*n* = 29).

**FIGURE 2:**
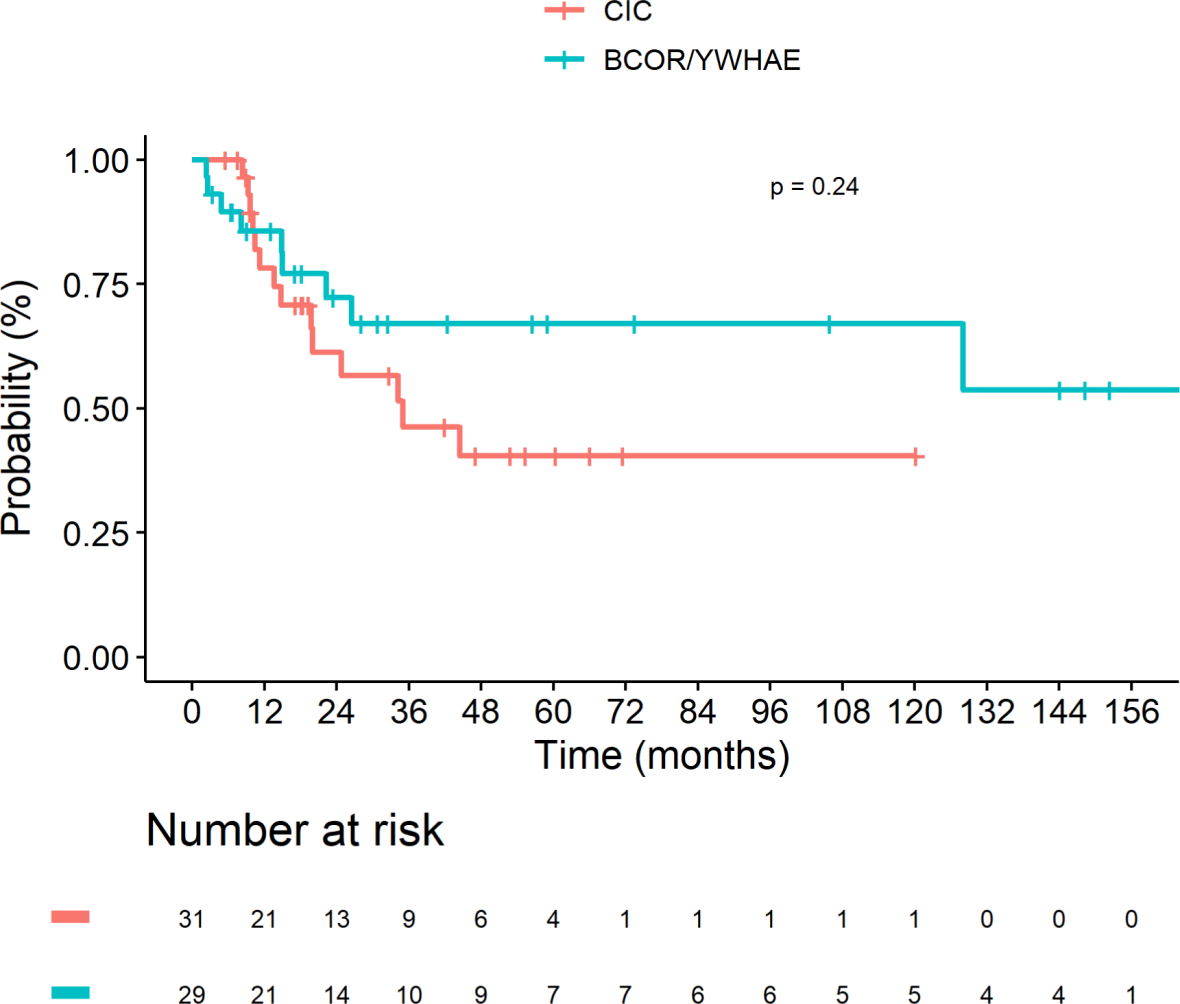
Kaplan–Meier estimates presenting OS of 60 patients with CIC‐fused sarcoma (*n* = 31) and BCOR rearranged sarcoma (*n* = 29).

Median follow‐up of the complete cohort was 47.1 months (range, 0.3–229.9), CI 95% [30.8–66.0]. Thirty‐two events occurred after a median delay of 8.2 months (range, 0.5–115): local progressive disease (*n* = 5), local relapse (*n* = 8), metastatic progressive disease (*n* = 10), metastatic relapse (*n* = 7), and combined progressive disease (*n* = 2). All deaths were due to tumor progression, but one patient had a chemotherapy‐related death. Among the 45 patients with initially localized disease, 30 (67%) patients were alive at the end of follow‐up: in CR (*n* = 22), with PR (*n* = 3), on treatment (*n* = 2) and with PD (*n* = 1) (tumor status unspecified, *n* = 2). Among the 15 patients with metastatic disease, seven patients (47%) were still alive: in CR (*n* = 5), in PR (*n* = 1) and on therapy (*n* = 1). Overall, the 3 year‐EFS were 44.0% (95% CI 28.7–67.5) and 41.2% (95% CI 25.4–67.0) for “*CIC* fused sarcoma*”* and “*BCOR* rearranged sarcoma” groups, respectively (*p* = 0.97). Three‐year OS were 46.3% (95% CI 29.6–72.4) and 67.1% (95% CI 50.4–89.3; *p* = 0.24), respectively. There was no significant difference of the 3‐year EFS and OS between the two groups (Figures 1 and 2).

### 3.5 Statistical analysis and prognostic factors

In term of clinical characteristics, age >13 years was the only clinical factor significantly associated with a better EFS in univariate and multivariate analysis (Table 4). Notably, these risk factors were only present in BCOR/YWHA tumors (*p* = 0.012) and not in CIC sarcomas (*p* = 0.84). Presence of metastases was the only factor significantly associated with a worse OS in univariate and multivariate analysis (Table 5; Figures S1 and S2). Males showed a significantly better OS in univariate analysis but not after adjustment for other risk factors (Table S3A,B).

**TABLE 4:** Risk factors in univariate and multivariate analysis for event‐free survival for the population with CIC and BCOR sarcomas.

Risk factors	Number of patients	Univariate				Multivariate		
		3y‐EFS % (95% CI)	HR	95% CI	*p* value	HR	95% CI	*p* value
Tumor type					0.98			0.94
CIC‐fused	31	44.0 (28.7–67.5)	1	[0.50; 1.95]		1		
BCOR rearranged	29	41.2 (25.4–67.0)	0.99			1.03	[0.44; 2.42]	
Median age					0.06			0.04
<13 yo	30	33.5 (18.8–59.9)	1			1		
>13 yo	30	51.9 (35.9–75.2)	0.52	[0.26; 1.03]		0.45	[0.21; 0.95]	
Gender					0.84			–
Female	26	43.2 (26.1–71.4)	1			NI	–	
Male	34	41.7 (27.1–64.3)	0.93	[0.47; 1.84]				
Tumor site					0.50			–
Head and neck	10	40.0 (18.7–85.5)	1			NI	–	
Limbs	18	36.1 (17.7–73.7)	0.62	[0.24; 1.65]				
Others	32	47.1 (31.5–70.6)	0.58	[0.24; 1.41]				
Tumor size					0.61			–
≤5 cm	16	57.4 (36.2–91.1)	1			NI	–	
>5 cm	42	40.6 (27.4–60.2)	1.23	[0.55; 2.75]				
T status					0.321			–
T1	21	46.3 (26.4–81.2)	1			NI	–	
T2	39	40.5 (27.1–60.5)	1.46	[0.68; 3.13]				
N status					0.28			–
N0	51	46.1 (33.4–63.6)	1			NI	–	
N1	6	50.0 (22.5–100.0)	0.92	[0.28; 3.07]				
Nx	3	33.3 (6.7–100.0)	3.05	[0.92; 10.1]				
M status					0.21			0.12
M0	45	46.6 (32.8–66.2)	1			1		
M1	15	29.3 (12.9–66.5)	1.64	[0.78; 3.41]		2.06	[0.83; 5.09]	
IRS stage					0.45			–
IRS I–II	10	43.8 (20.0–95.7)	1			NI	–	
IRS III	35	47.1 (31.6–70.1)	1.10	[0.41; 2.96]				
IRS IV	15	29.3 (12.9–66.5)	1.76	[0.60; 5.19]				

Abbreviations: EFS, event free survival; 95% CI, 95% confidential intervals; HR, Hazard Ratio; yo, year old; IRS‐I, complete resection; IRS‐II, microscopic incomplete resection; IRS‐III, macroscopic residue; IRS‐IV, presence of distant metastasis ; T, tumor; T1, tumor localized to tissue/organ of origin; T2, tumor beyong tissue/organ of origin; yo, year old; N0, absence of nodal involvment; N1, presence of nodal invovlment; Nx, data not specified;M0, localized tumor; M1, presence of distant metastasis.

**TABLE 5:** Risk factors in univariate and multivariate analysis for overall survival for the population with CIC and BCOR sarcomas.

Risk factors	Number of patients	Univariate				Multivariate		
		3y‐OS % (95% CI)	HR	95% CI	*p* value	HR	95% CI	*p* value
Tumor type					0.23			0.72
CIC‐fused	31	46.3 (29.6–72.4)	1			1		
BCOR rearranged	29	67.1 (50.4–89.3)	0.59	[0.25; 1.42]		1.23	[0.39; 3.92]	
Median age					0.92			–
<13 yo	30	49.8 (30.0–82.0)	1			NI	–	
>13 yo	30	57.2 (40.8–80.1)	0.96	[0.42; 2.18]				
Gender					0.24			0.16
Female	26	45.0 (26.1–77.3)	1			1		
Male	34	62.8 (47.2–83.4)	0.61	[0.27; 1.40]		0.52	[0.21; 1.31]	
Tumor site					0.67			–
Head and neck	10	61.7 (34.9–100.0)	1			NI	–	
Limbs	18	61.0 (40.6–91.7)	1.22	[0.31; 4.73]				
Others	32	52.2 (35.1–77.5)	1.63	[0.46; 5.73]				
Tumor size					0.63			–
≤5 cm	16	44.2 (21.1–92.8)	1			NI	–	
>5 cm	42	57.4 (42.8–77.1)	0.80	[0.33; 1.96]				
T status					0.24			0.53
T1	21	76.2 (58.1–99.9)	1			1		
T2	39	47.2 (32.3–69.0)	1.77	[0.65; 4.77]		1.39	[0.50; 3.88]	
N status					0.88			–
N0	51	57.4 (43.8–75.1)	1			NI	–	
N1	6	26.7 (5.1–100.0)	1.37	[0.40; 4.66]				
Nx	3	100 (100–100.0)	0.88	[0.12; 6.63]				
M status					0.04			0.07
M0	45	62.4 (47.7–81.7)	1			1		
M1	15	35.0 (15.5–78.8)	2.59	[1.08; 6.22]		2.88	[0.93; 8.97]	
IRS stage					0.07			–
IRS I–II	10	87.5 (67.3–100.0)	1			NI*	–	
IRS III	35	55.5 (38.9–79.1)	2.10	[0.47; 9.33]				
IRS IV	15	35.0 (15.5–78.8)	4.74	[1.01; 22.5]				

Abbreviations: CI, confidence interval; CT, chemotherapy; HR, hazard ratio; IRS, Intergroup Rhabdomyosarcoma Staging; M, metastasis; N, node; NI*, not introduced in multivariate model since IRS stage IV was equivalent to M1 status; NI, not introduced in multivariate model; R0, localized complete resection (IRS‐I); R1, microscopic incomplete resection (IRS‐II); R2, macroscopic residue (IRS III); Rx, data not available; T, tumor; yo, year old.

## 4 DISCUSSION

We reported the first clinical data of a European cohort of young patients with the novel genetically defined “*CIC* fused sarcomas” and “*BCOR* rearranged sarcomas” in soft tissues.

*“CIC* fused sarcomas” were described in a cohort of 115 patients as often found in soft tissue sarcoma (86%) and mainly occurring in adults (median age 32 years).^9^, ^22^ This analysis includes data on children, adolescents and young adults. The definition of young adults (AYA) varies and mostly includes patients up to 24 years. However, inclusion of data of older patients in cooperation with the adult oncologists are undoubtedly needed to get an overview on a larger cohort concerning age and this data do not reflect the overall age at diagnosis.

In our study, “*CIC* fused” STS were mainly located in the trunk and are often presenting with metastatic disease.^29^ In contrast, “*BCOR* rearranged sarcomas” are described as occurring more frequently in bone (64%) and in pediatric patients (76%)^30^: *BCOR::CCNB* was described more often in adolescents in contrast to *BCOR‐ITD*, more frequently occurring in infants.^30^, ^31^, ^32^ In our study, we cannot demonstrate any difference in the age of occurrence comparing *BCOR::CCNB* to other genetic alterations as *BCOR‐ITD* or the *YWHAE* alteration in contrast to data referring to all locations.^31^

Our cohort of patients harboring the *BCOR r*earrangement frequently had localized tumor, located in limbs and significantly younger at diagnosis than patients with “*CIC* fused” STS, confirming observations including locations in bone.^30^ Interestingly, we confirm that age was a significant factor for EFS, especially for BCOR/YWHA tumors, but not the biological entity itself. The precise reason to explain this difference is not totally clear. However, as this finding was only present in BCOR/YWHA sarcomas, one hypothesis could be that, in some way, therapy differences may influence the outcome of patients. Patients with BCOR tumors are more frequently younger and may have receive different therapy (i.e., less radiotherapy for instance) that may explain such difference.

These different genetic alterations seem to have a prognostic impact as patients with *BCOR* altered tumors are reported to have an overall poor prognosis. *BCOR::CCNB3* fused tumors are reported to have a 5‐year OS of 68%, in contrast to patients with *BCOR‐ITD* or *ZC3H7B::BCOR* tumors that have low survival rates, with more than 50% of patients in each group dying of relapse of their disease.^31^ In our experience we report a much higher number of IRS group IV tumors in the “*CIC* fused sarcomas” and only two in the “*BCOR* rearranged sarcomas” at diagnosis. Yet, the EFS of “*BCOR* rearranged sarcomas” as a group is lower than that of the “*CIC* fused sarcoma” group. This suggests that effective therapy was likely not delivered due to the younger age in the “*BCOR* rearranged sarcoma” group and also probably explains the higher salvage rate at relapse given the better overall survival compared to that seen in the “*CIC* fused sarcoma” group.

We recommend imaging guidelines developed for rhabdomyosarcoma as specific guidelines for *CIC* and *BCOR* sarcoma need to be developed.^33^ The initial extent of the disease and the patients age at the time of diagnosis might be responsible for the inhomogeneous treatment.^34^ The outcome of these patients is unsatisfactory with a 3‐year OS of 46.3% and 67.1%, respectively, but not statistically different (*p* = 0.24). These data correlate to the rare published data on these patient groups including bone primary: 5‐year OS 43% for “*CIC* fused sarcoma” and 72% for “*BCOR* rearranged sarcoma”.^22^, ^35^ As in other STS, metastatic status was a clear prognostic factor for OS.

We are able to contribute detailed data on CHT and local treatment: Patients of both entities were more likely to be treated in Europe according to an STS protocol as (non‐) rhabdomyosarcoma, rather than an Ewing one. However, the main drugs are similar in these protocols and the main difference lies on the total number of CHT courses. Multimodal strategy seems important but larger cohort are needed to evaluate the value of each therapy in such patients.

The main weaknesses of these data are their retrospective character, extracted from different databases or centers. As a limitation, the timing of the testing could not be evaluated. However, as patients are included diagnosed before 2015, few patients were included with the molecular diagnosis made some years after initial histologic diagnosis. The frequency of these biologically distinct cancers is extremely rare. Considering CWS patients from the European countries Germany, Switzerland, Austria, Poland, Sweden, and Finland, 11 patients with *CIC‐*fused*/BCOR* rearranged STS of the total cohort of 247 patients with the histologic diagnosis of USRCC/UDS/ES of STS irrespective of the molecular diagnosis were registered in the European CWS studies and the STS registry SoTiSaR in this time. As further limitation we are not able to report on numbers of patients with “*CIC* fused sarcoma” and “*BCOR* rearranged sarcoma” in bone, kidney, and CNS from all the institutions. To get a sense of the frequency and clinical data of these biologically distinct cancers larger international retrospective studies including studies and registries of bone, kidney, and CNS are necessary.

An international common strategy based on the somatic genetic background is undoubtedly required and a new international risk stratification considering the overall unfavorable prognostic of these entities needs to be built up.

These data also stress the need to perform additional molecular testing in case of any tumor with Ewing characteristics that do not harbor the canonic EWS fusion transcript.^36^ Tumor genomic sequencing and assignment of genotype‐based groups should be incorporated to help understanding of different clinical outcomes.^37^ Finally, new treatment options need to be developed. Since some of them start to seem promising, international collaborations, to develop trials including new treatment strategies, are of highest importance in these rare diseases.^38^, ^39^ Applying a distinctive signature of 537 deregulated genes in CIC‐rearranged sarcoma with RNA seq, Palmirini et al. in a recent paper showed that the *CIC::DUX4* transcriptional profile compared that of ES was enriched in gene sets including DUSP4, ETV1/4, IGF2, and IGF2BPs, PTX 1 and 3 that are associated with activity of irinotecan, proteasome inhibitors (bortezomib and MG262), and histone deacetylase inhibitors (scriptaid and vorinostat).^40^ Future common analyses on patients with these rare diseases occurring in bone and soft tissue sarcoma are needed to better compare different treatment regimen, to define prognostic factors and to develop new treatment strategies.

## AUTHOR CONTRIBUTIONS

**Monika Sparber‐Sauer:** Conceptualization (lead); data curation (equal); formal analysis (equal); resources (equal); validation (equal); writing – original draft (equal); writing – review and editing (lead). **Nadege Corradini:** Resources (equal); visualization (equal); writing – original draft (equal); writing – review and editing (equal). **Maria Carmen Affinita:** Resources (equal); writing – original draft (equal); writing – review and editing (equal). **Giuseppe Maria Milano:** Resources (equal); writing – original draft (equal); writing – review and editing (equal). **Gaëlle Pierron:** Formal analysis (equal); methodology (equal); resources (equal); writing – original draft (equal); writing – review and editing (equal). **Matthieu Carton:** Data curation (lead); formal analysis (lead); methodology (lead); resources (equal); software (equal); supervision (equal); validation (equal); writing – original draft (equal); writing – review and editing (equal). **Franck Tirode:** Resources (equal); visualization (equal); writing – original draft (equal); writing – review and editing (equal). **Daniel Pissaloux:** Resources (equal); writing – original draft (equal); writing – review and editing (equal). **Rita Alaggio:** Resources (equal); validation (equal); writing – review and editing (equal). **Christian Vokuhl:** Resources (equal); validation (equal); writing – review and editing (equal). **Gianni Bisogno:** Resources (equal); validation (equal); writing – review and editing (equal). **Pablo Berlanga:** Resources (equal); validation (equal); writing – review and editing (equal). **Andrea Ferrari:** Conceptualization (equal); data curation (equal); methodology (equal); project administration (equal); resources (equal); validation (equal); visualization (equal); writing – review and editing (equal). **Daniel Orbach:** Conceptualization (lead); data curation (lead); formal analysis (lead); funding acquisition (lead); investigation (equal); methodology (equal); project administration (equal); resources (equal); supervision (lead); validation (equal); visualization (equal); writing – original draft (lead); writing – review and editing (lead).

## FUNDING INFORMATION

The Soft Tissue Sarcoma Registry (SoTiSaR) was supported by the Deutsche Kinderkrebsstiftung, Bonn, Germany, grant no. A2007/13DKS2009.08 and by the Foerderkreis Krebskranke Kinder Stuttgart, Germany. The data collection of Padua has been supported by Fondazione Città della Speranza, Padova, Italy. The authors wish to thank S Wisna for the financial help.

## CONFLICT OF INTEREST STATEMENT

The authors declare that they have no known competing financial interests or personal relationships that could have appeared to influence the work reported in this paper.

## CONSENT STATEMENT

Informed consent has been obtained from all CWS and participating patients and/or their parents/guardians according to the legal requirements.

## Supporting information


Figure S1.

Figure S2.

Tables S1–S3.

## Data Availability

Not applicable.
