# Supplementary material for: Clinical characteristics and outcomes for children, adolescents and young adults with “CIC‐fused” or “BCOR‐rearranged” soft tissue sarcomas: A multi‐institutional European retrospective analysis
Source: Cancer Med. 2023 May 22;12(13):14346–59. doi: 10.1002/cam4.6113 (PMC10358194; doi:10.1002/cam4.6113)

**Figure S1**. Kaplan-Meier estimates presenting OS for patients according to presence of metastases.


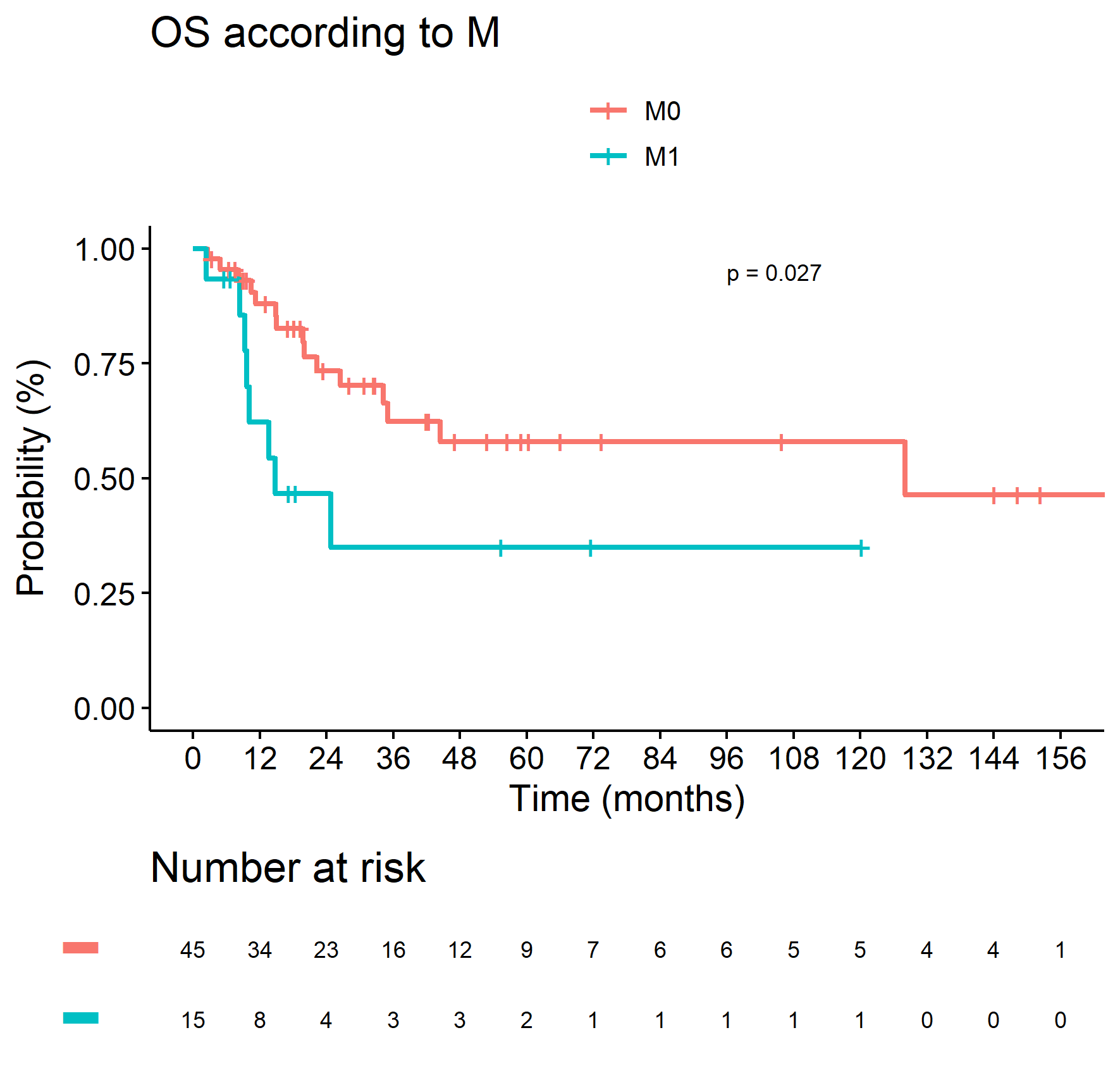

Supplement: Supplementary file 1 — Figure S1. [file CAM4-12-14346-s001.docx]
