# Supplementary material for: Clinical characteristics and outcomes for children, adolescents and young adults with “CIC‐fused” or “BCOR‐rearranged” soft tissue sarcomas: A multi‐institutional European retrospective analysis
Source: Cancer Med. 2023 May 22;12(13):14346–59. doi: 10.1002/cam4.6113 (PMC10358194; doi:10.1002/cam4.6113)

**Figure S2**. Kaplan-Meier estimates presenting OS for patients CIC (A) and BCOR (B) fused sarcomas according to IRS.

**A**


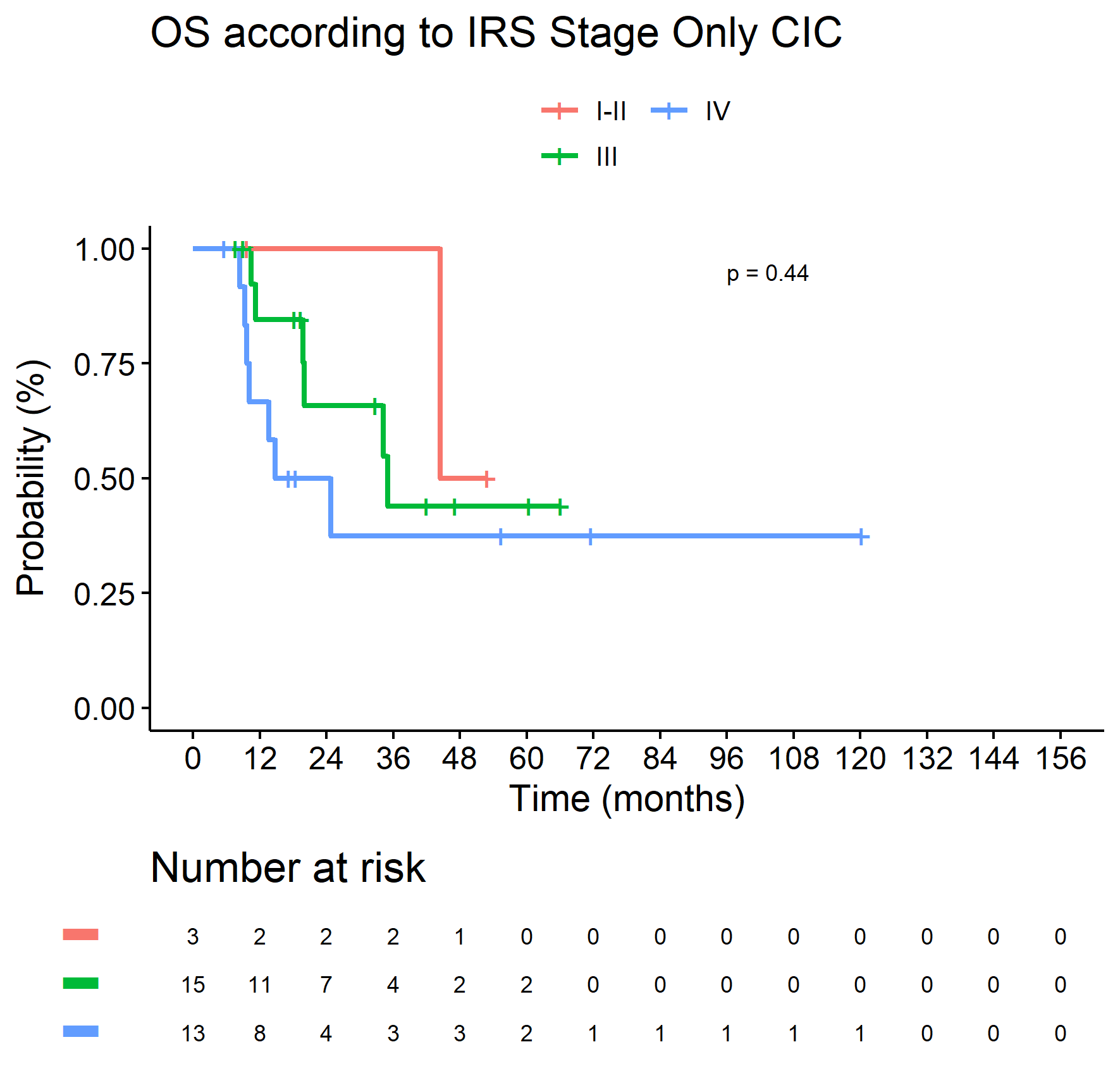


B


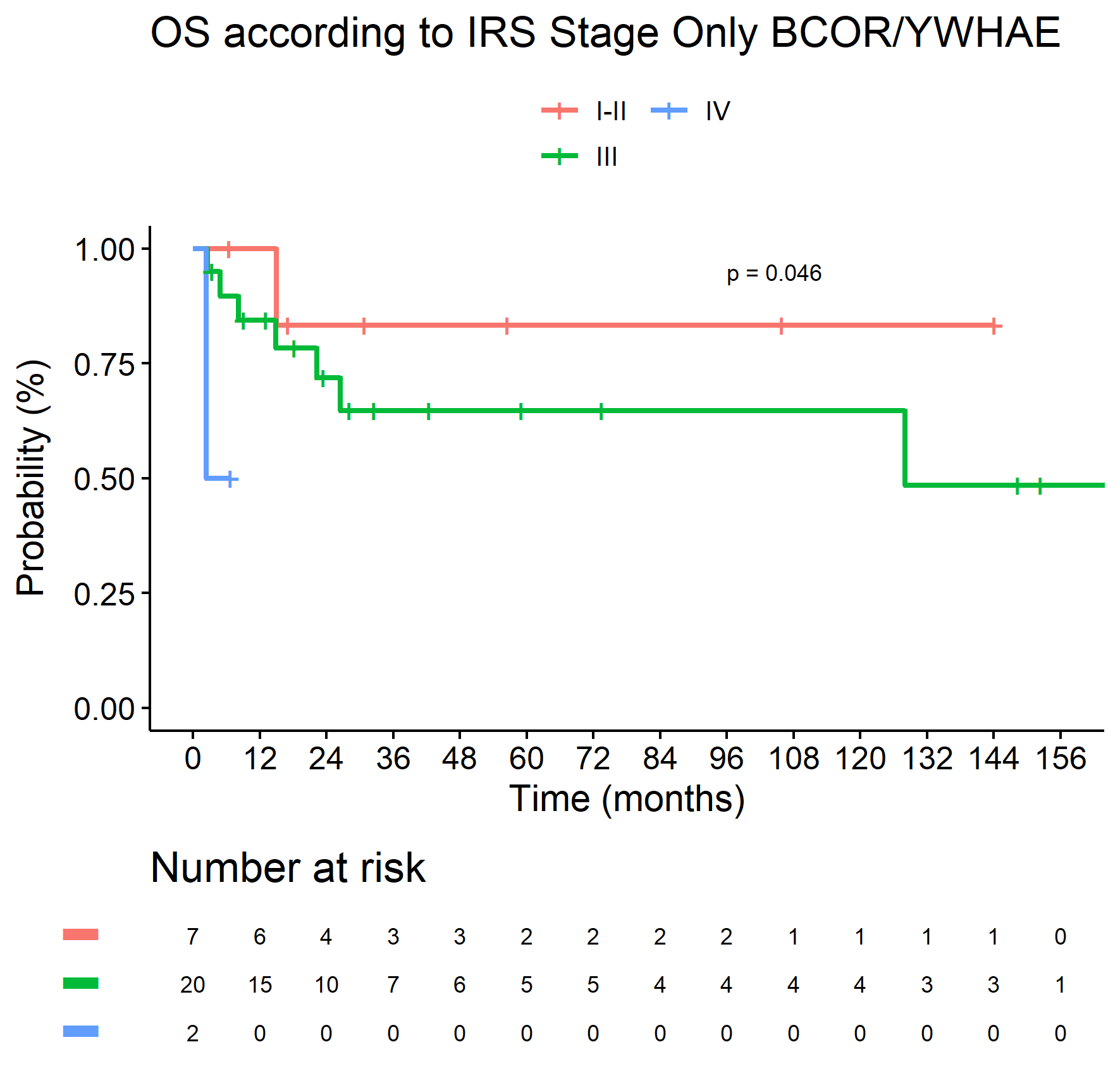

Supplement: Supplementary file 2 — Figure S2. [file CAM4-12-14346-s002.docx]
