# Supplementary material for: Clinical characteristics and outcomes for children, adolescents and young adults with “CIC‐fused” or “BCOR‐rearranged” soft tissue sarcomas: A multi‐institutional European retrospective analysis
Source: Cancer Med. 2023 May 22;12(13):14346–59. doi: 10.1002/cam4.6113 (PMC10358194; doi:10.1002/cam4.6113)
Supplement: Supplementary file 3 — Tables S1–S3. [file CAM4-12-14346-s003.docx]

**Table S1**. Clinical characteristics of patients with BCOR rearranged tumors

|  |  | *BCOR::CCNB3*  N=18 (%) | BCOR-ITD  N=7 (%) | *MAML::BCOR*  N=1 (%) | *YWHAE::NUTM2B*  N=3 (%) | Total  N=29 (%) | P value |
| --- | --- | --- | --- | --- | --- | --- | --- |
| Sex | Female | 4 (22.2%) | 1 (14.3%) | 0 (0%) | 1 (33.3%) | 6 (20.7%) | 1 |
|  | Male | 14 (77.8%) | 6 (85.7%) | 1 (100%) | 2 (66.7%) | 23 (79.3%) |  |
| Age (median) | <13yo | 12 (66.7%) | 6 (85.7%) | 0 (0%) | 3 (100%) | 21 (72.4%) | 0.27 |
|  | >13yo | 6 (33.3%) | 1 (14.3%) | 1 (100%) | 0 (0%) | 8 (27.6%) |  |
| Tumor site | Head and neck | 2 (11.1%) | 2 (28.6%) | 0 (0%) | 0 (0%) | 4 (13.8%) | 0.74 |
|  | Limbs | 4 (22.2%) | 0 (0%) | 0 (0%) | 0 (0%) | 4 (13.8%) |  |
|  | Others | 12 (66.7%) | 5 (71.4%) | 1 (100%) | 3 (100%) | 21 (72.4%) |  |
| Tumor size | <5 cm | 6 (33.3%) | 2 (28.6%) | 0 (0%) | 0 (0%) | 8 (27.6%) | 0.88 |
|  | >5 cm | 12 (66.7%) | 5 (71.4%) | 1 (100%) | 3 (100%) | 21 (72.4%) |  |
| T | T1 | 6 (33.3%) | 4 (57.1%) | 0 (0%) | 1 (33.3%) | 11 (37.9%) | 0.7 |
|  | T2 | 12 (66.7%) | 3 (42.9%) | 1 (100%) | 2 (66.7%) | 18 (62.1%) |  |
| N | N0 | 16 (88.9%) | 7 (100%) | 0 (0%) | 2 (66.7%) | 25 (86.2%) | 0.061 |
|  | N1 | 2 (11.1%) | 0 (0%) | 1 (100%) | 0 (0%) | 3 (10.3%) |  |
|  | NX | 0 (0%) | 0 (0%) | 0 (0%) | 1 (33.3%) | 1 (3.4%) |  |
| M | M0 | 18 (100%) | 7 (100%) | 1 (100%) | 1 (33.3%) | 27 (93.1%) | 0.014 |
|  | M1 | 0 (0%) | 0 (0%) | 0 (0%) | 2 (66.7%) | 2 (6.9%) |  |
| IRS Stage | I-II | 5 (27.8%) | 2 (28.6%) | 0 (0%) | 0 (0%) | 7 (24.1%) | 0.073 |
|  | III | 13 (72.2%) | 5 (71.4%) | 1 (100%) | 1 (33.3%) | 20 (69%) |  |
|  | IV | 0 (0%) | 0 (0%) | 0 (0%) | 2 (66.7%) | 2 (6.9%) |  |

**Abbreviations**: yo, year old, T: tumor, N: Node, M: Metastasis, IRS: Intergroup Rhabdomyosarcoma Staging

**Table S2**. Clinical characteristics of the patients with CIC fused tumors.

|  |  | CIC-DUX 4  N=22 (%) | CIC break positive  N=7 (%) | ATXN1/NUTM1  N=2 (%) | Total | P value |
| --- | --- | --- | --- | --- | --- | --- |
| Sex | Female | 14 (63.6%) | 4 (57.1%) | 2 (100%) | 20 (64.5%) | 0.72 |
|  | Male | 8 (36.4%) | 3 (42.9%) | 0 (0%) | 11 (35.5%) |  |
| Age (median) | <13yo | 3 (13.6%) | 4 (57.1%) | 2 (100%) | 9 (29%) | 0.003 |
|  | >13yo | 19 (86.4%) | 3 (42.9%) | 0 (0%) | 22 (71%) |  |
| Tumor site | HN | 3 (13.6%) | 1 (14.3%) | 2 (100%) | 6 (19.4%) | 0.16 |
|  | Limbs | 11 (50%) | 3 (42.9%) | 0 (0%) | 14 (45.2%) |  |
|  | Others | 8 (36.4%) | 3 (42.9%) | 0 (0%) | 11 (35.5%) |  |
| Tumor size | <5 cm | 5 (23.8%) | 3 (42.9%) | 0 (0%) | 8 (27.6%) | 0.53 |
|  | >5 cm | 16 (76.2%) | 4 (57.1%) | 1 (100%) | 21 (72.4%) |  |
|  | NA | 1 | 0 | 1 | 2 |  |
| T | T1 | 7 (31.8%) | 3 (42.9%) | 0 (0%) | 10 (32.3%) | 0.69 |
|  | T2 | 15 (68.2%) | 4 (57.1%) | 2 (100%) | 21 (67.7%) |  |
| N | N0 | 19 (86.4%) | 6 (85.7%) | 1 (50%) | 26 (83.9%) | 0.32 |
|  | N1 | 2 (9.1%) | 0 (0%) | 1 (50%) | 3 (9.7%) |  |
|  | NX | 1 (4.5%) | 1 (14.3%) | 0 (0%) | 2 (6.5%) |  |
| M | M0 | 12 (54.5%) | 4 (57.1%) | 2 (100%) | 18 (58.1%) | 0.69 |
|  | M1 | 10 (45.5%) | 3 (42.9%) | 0 (0%) | 13 (41.9%) |  |
| IRS Stage | I-II | 2 (9.1%) | 0 (0%) | 1 (50%) | 3 (9.7%) | 0.43 |
|  | III | 10 (45.5%) | 4 (57.1%) | 1 (50%) | 15 (48.4%) |  |
|  | IV | 10 (45.5%) | 3 (42.9%) | 0 (0%) | 13 (41.9%) |  |

**Table S3A,B**. Univariate and multivariate analysis for the population with localized tumors

**Table S3A** – Event free survival

| Risk factors | Univariate | | | | Multivariate | | |
| --- | --- | --- | --- | --- | --- | --- | --- |
|  | **Number of patients** | **HR** | **95% CI** | **P Value** | **HR** | **95% CI** | **P Value** |
| Tumor type:   - CIC fused - BCOR rearranged | 18  27 | 1  0.98 | [0.42; 2.27] | 0.953 | 1  0.92 | [0.43; 1.99] | 0.837 |
| Median age:   - < 13 yo - > 13 yo | 25  20 | 1  0.36 | [0.15; 0.88] | 0.018 | 1  0.44 | [0.21; 0.92] | 0.027 |
| Gender:   - Female - Male | 20  25 | 1  0.74 | [0.33; 1.65] | 0.465 | NI | - | - |
| Tumor site:   - Head and Neck - Limbs - Others | 8  15  22 | 1  0.45  0.33 | [0.15; 1.31]  [0.12; 0.94] | 0.145 | 1  0.74  0.48 | [0.27; 2.09]  [0.19; 1.22] | 0.300 |
| Tumor size:  - ≤ 5cm  - > 5 cm | 14  29 | 1  1.07 | [0.43; 2.64] | 0.883 | 1  0.57 | [0.20; 1.65] | 0.309 |
| T status   - T1 - T2 | 18  27 | 1  1.99 | [0.79; 5.03] | 0.127 | 1  1.69 | [0.73; 3.92] | 0.211 |
| N status:   - N0 - N1 - Nx | 38  5  2 | 1  0.73  2.64 | [0.17; 3.18]  [0.61; 11.46] | 0.456 | NI | - | - |
| IRS stage:   - IRS I-II - IRS III | 10  35 | 1  1.16 | [0.43; 3.13] | 0.76 | NI | - | - |

**Table S3B** – Overall survival

| Risk factors | Univariate | | | | Multivariate | | |  |
| --- | --- | --- | --- | --- | --- | --- | --- | --- |
|  | **Number of patients** | **HR** | **95% CI** | **P Value** | **HR** | **95% CI** | **P Value** |  |
| Tumor type:   - CIC fused - BCOR rearranged | 18  27 | 1  0.67 | [0.23; 1.91] | 0.455 | 1  0.83 | [0.21; 3.21] | 0.79 |  |
| Median age:   - < 13 yo - > 13 yo | 25  20 | 1  0.53 | [0.18; 1.56] | 0.237 | 1  0.50 | [0.15; 1.61] | 0.24 |  |
| Gender:   - Female - Male | 20  25 | 1  0.34 | [0.11; 1.01] | 0.043 | 1  0.38 | [0.10; 1.41] | 0.24 |  |
| Tumor site:   - Head and Neck - Limbs - Others | 8  15  22 | 1  0.78  0.86 | [0.19; 3.28]  [0.22; 3.35] | 0.946 | NI | - | - |  |
| Tumor size:  - ≤ 5cm  - > 5 cm | 14  29 | 1  0.57 | [0.20; 1.65] | 0.309 | NI | - | - |  |
| T status   - T1 - T2 | 18  27 | 1  2.44 | [0.68; 8.68] | 0.137 | 1  2.29 | [0.63; 8.29] | 0.21 |  |
| N status:   - N0 - N1 - Nx | 38  5  2 | 1  1.48  1.2 | [0.33; 6.72]  [0.15; 9.27] | 0.880 | NI | - | - |  |
| IRS stage:   - IRS I-II - IRS III | 10  35 | 1  2.14 | [0.48; 9.53] | 0.274 | NI | - | - |  |

**Abbreviations**: yo, year old, T: tumor, N: Node, M: Metastasis, IRS: Intergroup Rhabdomyosarcoma Staging, R0, complete resection (IRS-I); R1, microscopic resection (IRS-II); R2, macroscopic residue (IRS III); Rx, data not available; NI, not introduced in multivariate model; CI, Confidence interval.
